# Exploring the role of NLRP3 infalmmasome in diabetes: a literature review and bibliometric analysis

**DOI:** 10.3389/fendo.2024.1443798

**Published:** 2024-12-09

**Authors:** Yi Tan, Shaotao Chen, Tianjiao Gao, Sixian Wang, Xinfeng Zhou, Mingjun Liu

**Affiliations:** ^1^ Departments of Acupuncture and Massage, Changchun University of Chinese Medicine, Changchun, Jilin, China; ^2^ Office of Scientific Research, Affiliated Hospital to Changchun University of Chinese Medicine, Changchun, Jilin, China

**Keywords:** NLRP3 infalmmasome, diabetes, bibliometric analysis, VOSviewer, Citespace

## Abstract

**Background:**

Diabetes has emerged as the foremost public health challenge of the 21st century, with a notable shift towards managing it through an inflammatory lens. This study seeks to investigate the role of NLRP3 infalmmasome in diabetes over the past ten years, leveraging bibliometric analysis to pinpoint prevailing trends, underscore critical focal points, and establish a roadmap for subsequent research endeavors.

**Method:**

A literature search was conducted based on the SCI-E database, and all recorded results were downloaded in plain text format for subsequent analysis. The analysis was carried out using Vosviewer1.6.18, citespace6.3R1, and Microsoft Excel 2021 software, focusing on the following terms: country, institution, author, journal, references, and keywords.

**Results:**

From January 1, 2014, to December 31, 2023, a total of 1373 articles were retrieved, with China, the United States, and Italy contributing the majority of records. Harbin Medical University, Nanjing Medical University, and Central South University stand as the top three most productive institutions. “International Journal of Molecular Sciences” leads the way with the highest number of publications, closely followed by “Frontiers in Immunology” and “Frontiers in Pharmacology.” Authors Wang Wei boast the most publications, closely followed by Li Xiang and Wang Yan. Within the superimposed keyword network, four primary clusters emerge: (1) exploring the link between NLRP3 infalmmasome and inflammatory diseases like diabetes; (2) investigating the cellular-level pathogenesis of diabetes-related conditions; (3) examining diabetes characteristics and associated suppression techniques; (4) studying cell morphology alterations, including pyroptosis. Over the past five years, key topics in this field have revolved around the “heart”, “damage”, “caspase 1 activation”, “NLRP3”, and “diabetic kidney disease”.

**Conclusion:**

This paper has identified the hot spots and trends concerning the role of NLRP3 infalmmasome in diabetes, thereby providing a valuable reference for future research. Furthermore, it is anticipated that pyroptosis and diabetes-related diseases will become frontier research topics that may garner significant attention in the coming years.

## 1 Introduction

Diabetes mellitus (DM) represents a group of progressive disorders marked by hyperglycemia, posing a significant threat to human health and imposing a substantial burden on society. The exponential rise in DM prevalence stands as one of the most critical global health crises of the 21st century. In 2009, 285 million people had diabetes (1), increasing to 366 million in 2011 (2), 382 million in 2013 (3), 415 million in 2015 (4) and 425 million in 2017 (5). In 2019, approximately 463 million people will have diabetes, representing 9.3 per cent of the global adult population (aged 20-79 years) (6). As of 2021, the global count of adult diabetics stands at 537 million (10.5%), 15.9827 per cent over 2019,with a concerning statistic revealing that approximately 44.7% of these adults (equating to 240 million individuals) remain undiagnosed. The number of patients is projected to rise to 578 million by 2030 and is expected to reach 784 million by 2045 (7). The rapid growth of DM has become a major challenge in global public health, leading to increased complications (8, 9), increased economic burden, and increased need for public health interventions (10) while affecting work productivity. The DM epidemic is therefore not only a health issue, but also a major economic and social challenge. Collaborative efforts are needed to scale up DM prevention and control measures globally in order to mitigate the public health impact of the epidemic.Notably, chronic non-infectious inflammation plays a pivotal role in both the onset and progression of DM (11).

Recent evidence has demonstrated that the Nod-like receptor protein 3 inflammasome(NLRP3 inflammasome) plays a critical role in regulating endoplasmic reticulum stress-induced impairments in glucose tolerance, insulin resistance, inflammatory cell apoptosis, and endothelial dysfunction within adipose tissue (12). The NLRP3 infalmmasome, a protein complex, regulates the innate immune response through the activation of caspase-1 and the inflammatory cytokines interleukin (IL)-1β and IL-18 (13). These cytokines play a key role in promoting the inflammatory response and have the potential to amplify the inflammatory effects, thereby exacerbating insulin resistance and β-cell dysfunction, which are key features of both type 1 diabetes and type 2 diabetes mellitus (T2DM) (14). The deregulated activation of NLRP3 infalmmasome has been linked to the development of various inflammasome-related diseases, underscoring its significance as a primary target for the treatment of inflammatory diseases (15). Thus far, the role of NLRP3 infalmmasome in DM has undergone extensive examination, yielding notable advancements that hold substantial importance for DM treatment. Nonetheless, to date, no comprehensive and objective report has been made on the progression of relevant publishing trends, influential authors or institutions, their collaborations, the knowledge base, and emerging hot spots.

In this study, the bibliometric software tools CiteSpace and VOSviewer were utilized to provide an objective depiction of the knowledge base and emerging trends concerning the role of NLRP3 infalmmasome in DM over the past decade. Initially, fundamental data was quantified and presented through an analysis encompassing annual publication trends, contributing countries and regions, institutions, authors, co-cited authors, and journals. Subsequently, by examining co-citation contributions, we gained deeper insights into the underlying knowledge base. Lastly, we dynamically observed hotspots and trends by exploring co-citation literature emergence, keyword co-occurrence, and keyword emergence.

## 2 Materials and methods

### 2.1 Data collection

**Figure 1:**
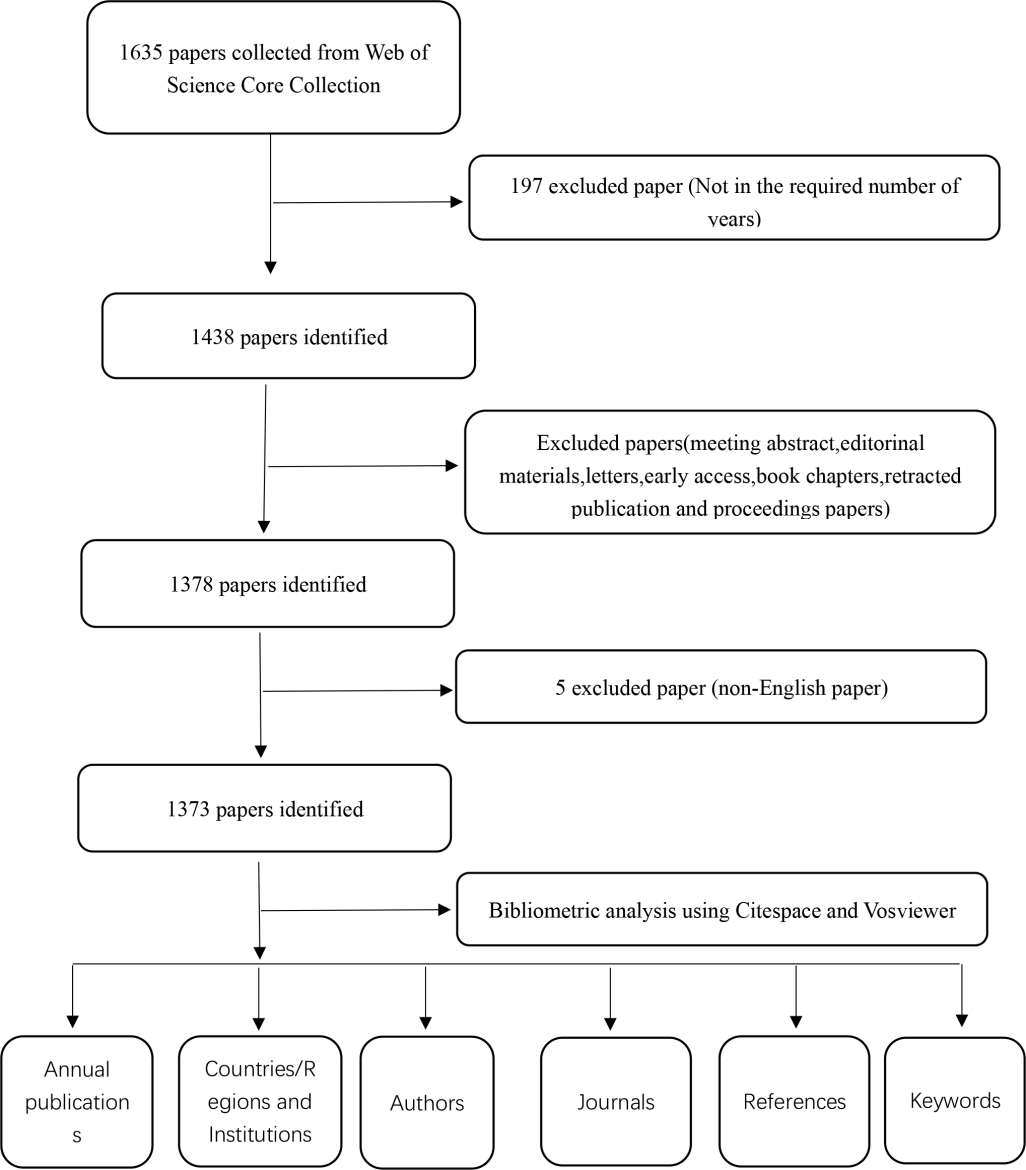
The figure presents a one-frame flow chart that illustrates the comprehensive criteria and the step-by-step process of conducting a bibliometric analysis on publications chosen from the WOS database, specifically exploring the role of NLRP3 infalmmasome in diabetes.

On May 17, 2024, an exhaustive search was conducted on the Web of Science Core Collection (WoSCC) to retrieve all citations published between January 1, 2014, and December 31, 2023. The search was focused on the following topics: TS=[”NLRP3 inflammasome” (Topic) OR “NLRP3” (Topic) OR “NLR Family, Pyrin Domain-Containing 3 Protein “ (Topic) OR” NACHT, LRR and PYD Domains-Containing Protein 3” (Topic) OR “NACHT, LRR and PYD Domains Containing Protein 3” (Topic) OR “NLRP3 Protein “ (Topic)] AND TS=[“Diabetes*” (Topic) OR “Diabetes mellitus*” (Topic)]. The search parameters were set to include only articles and reviews written in English, excluding conference abstracts, preemptive experience articles, edited materials, letters, collections, anthologies, news projects, book chapters, hardware reviews, and withdrawn publications. As a result, 1373 articles were carefully selected, consisting of 928 articles and 445 reviews. From each of these publications, comprehensive data was extracted, including the title, year of publication, country or region, institution, author, journal, references, and key words. For a detailed overview of the literature extraction process, refer to **Figure 1**.

### 2.2 Data analysis

For bibliometric and knowledge mapping analysis, we utilized CiteSpace 6.3R1, vosviewer 1.6.18, Scimago graphica, and Microsoft Excel 2021.

Vosviewer has the capability to construct maps of authors or journals utilizing co-citation data, and to create keyword maps based on co-occurrence data (16). In this article, we employ it for examining co-occurrences of executive agencies, authors and co-cited authors, journals and co-cited journals, as well as keywords. Additionally, it is utilized in conjunction with Scimago graphica to explore co-occurrences of countries/regions. Scimago graphica, which debuted in May 2021, stands as the most recent software for effortless dataset browsing, filtering, and visualization, featuring intuitive drag-and-drop functionality. As a code-free tool, it offers ease of use and broad applicability.

CiteSpace is a software designed for the visual analysis and exploration of academic literature within various research fields or disciplines (17, 18). This paper utilizes the co-cited literature, the emergence of co-cited literature, and keyword emergence to gain a deeper understanding of the field’s introduction and developments.

This article employs Microsoft Excel 2021 to examine annual publication trends while simultaneously searching for the impact index of magazines and periodicals in 2022 within the Web of Science.

## 3 Results

### 3.1 Relevant publication information

**Figure 2:**
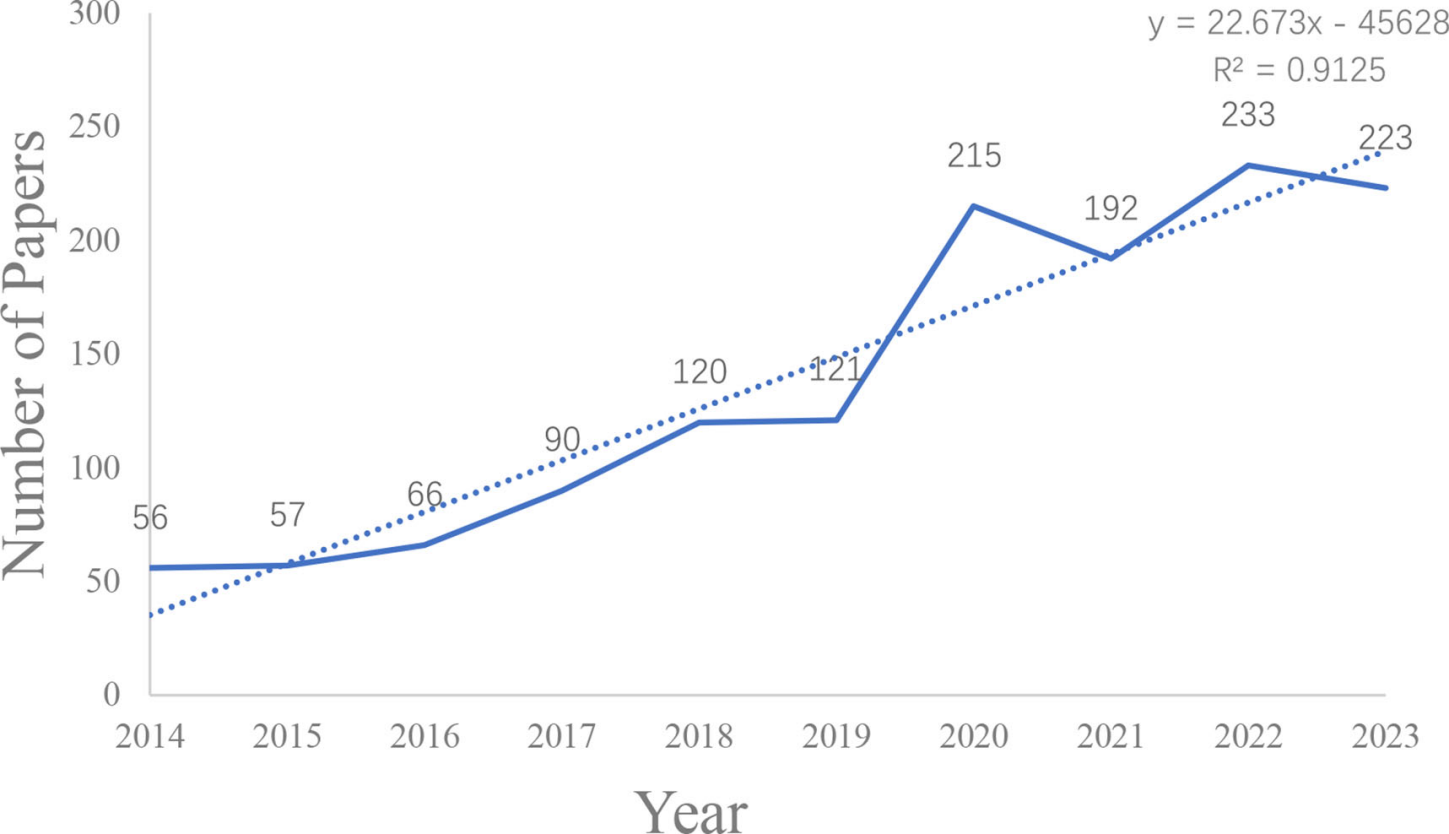
Distribution trend from January 1, 2014, to December 31, 2023.

The annual number of publications holds great significance in the study of this field, as it serves as an indicator of the field’s knowledge development to a certain extent. Referring to **Figure 2**, we observe the published volume on NLRP3 infalmmasome in DM over the past decade. Between 2014 and 2018, the published volume remained low with slow growth. However, 2019 emerged as a notable turning point, marking a rapid surge in the number of articles published, culminating in a historic high in 2022. To gain deeper insights into the output trend, a linear trend line for annual publications was established, yielding the equation y=22.673x-45628, where Y denotes annual publications and X represents the year. The model’s coefficient of determination (R²) stands at 0.9125. Overall, there has been a steady increase in knowledge pertaining to the role of NLRP3 infalmmasome in DM, reflecting the growing popularity and significance of this subject matter.

### 3.2 Distribution of countries/regions

**Figure 3:**
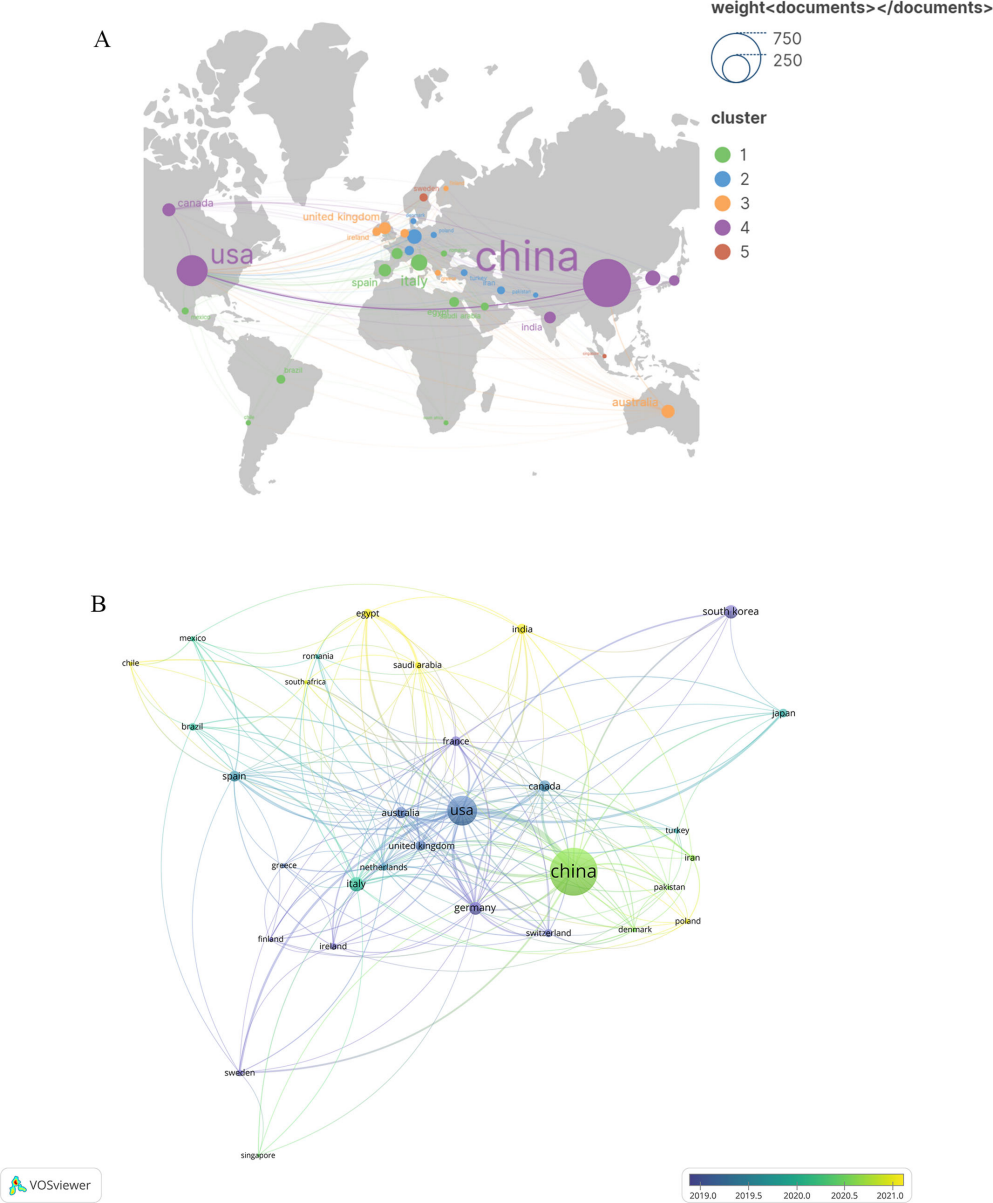
A visualization map of countries. **(A)** Network diagram of the top 35 countries. **(B)** Dynamics and trends of the top 35. The total linkstrength :540.Items:31.Clusters:5.Links:203.

A total of 78 countries have delved into this topic. **Figure 3** illustrates the citation frequency and collaboration among the top 35 countries based on their publication count. Specifically, **Figure 3A** presents the number of publications and the co-occurrence between various countries. On the other hand, **Figure 3B** explores the dynamics and trends, with countries represented by circles—the larger the circle, the higher the number of documents produced by that country. The lines connecting two nodes indicate that those two countries are mentioned together in a document. **Table 1** highlights the top 10 countries engaged in this thematic study. Notably, China leads with the highest number of publications (657), followed by the United States(273)and Italy (75). In terms of citations, China has garnered 21,860, surpassing the United States’ (20,615) and Italy’s (4114).

**Table 1:** Top 10 most productive countries/regions with publications on the Role of NLRP3 infalmmasome in Diabetes from 2014 to 2023.

Rank	Counties/Regions	Documents(n)	Ciations(n)	Average ciatations(n)	Total link strength
1	China	657	21860	33.2725	121
2	USA	273	20615	75.5128	204
3	Italy	75	4114	54.8533	42
4	South korea	64	2239	34.9844	14
5	Germany	60	6548	109.1333	76
6	Australia	49	5152	105.1429	63
7	Canada	48	1941	40.4375	48
8	Spain	44	2168	49.2727	39
9	United kingdom	40	1776	44.4	42
10	India	39	693	17.7692	17

### 3.3 University and institutional

**Figure 4:**
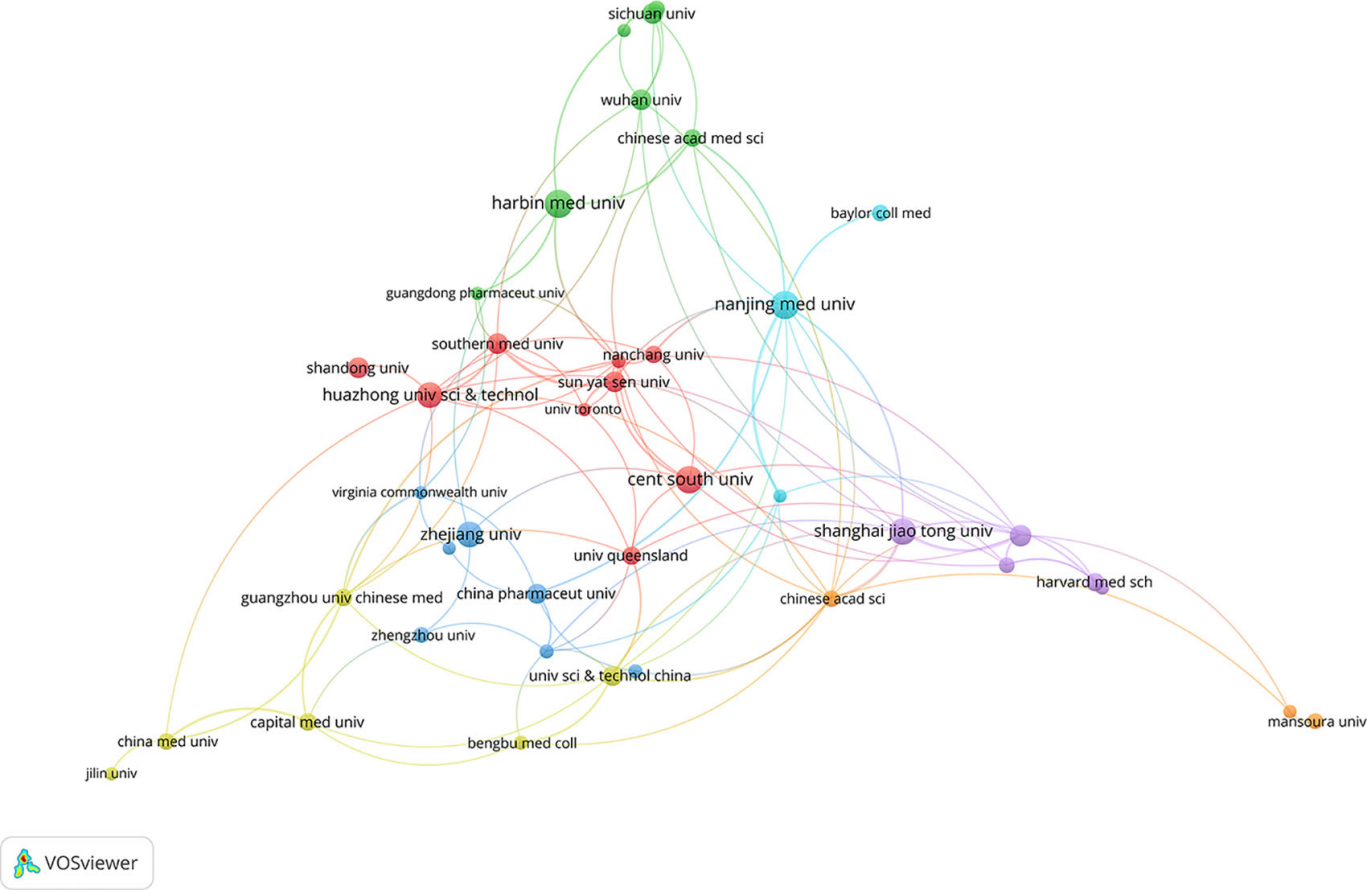
A visualization map of institutions.The total linkstrength :148.Items:40.Clusters:7.Links:110.

A total of 1780 institutions contributed to the publication of relevant articles on this topic. **Table 2** highlights the top 15 institutions with over 5 relevant publications each. Notably, Harbin Medical University leads the pack, followed by Nanjing Medical University, Central South University, Shanghai Jiao Tong University, Huazhong University of Science and Technology, and Zhejiang University. Collectively, these top 15 institutions published 291 articles, constituting 21.19% of the total publications. Among them, the University of Science and Technology of China garnered the highest number of citations. **Figure 4** delves into the collaboration amongst literature-publishing organizations. Using Vosviewer, with a minimum occurrence threshold of 10, we identified 44 prominent institutions. An analysis based on total contact strength placed Nanjing Medical University (n=19), Shanghai Jiao Tong University (n=17), Jinan University (n=16), Chinese Academy of Sciences (n=15), and the University of Science and Technology of China (n=14) in the top five positions.

**Table 2:** Top 15 institutions with publications on the Role of NLRP3 infalmmasome in Diabetes from 2014 to 2023.

Rank	Counties/Regions	Documents(n)	Ciations (n)	Average ciatations (n)	Total link strength
1	Harbin Medical University	25	1113	44.52	9
2	Nanjing Medical University	25	536	21.44	19
3	Central South University	24	558	23.25	8
4	Shanghai Jiao tong University	23	1084	47.1304	17
5	Huazhong University Of Science And Technology	22	469	21.3182	11
6	Zhejiang University	22	1699	77.2273	4
7	Fudan University	18	641	35.6111	14
8	Shandong University	17	519	30.5294	1
9	Sichuan University	17	437	25.7059	4
10	Southern Medical University	17	485	28.5294	12
11	Sun Yat-sen University	17	488	28.7059	13
12	Wuhan University	17	832	48.9412	7
13	China Pharmaceutical University	16	577	36.0625	7
14	University of Science and Technology of China	16	3099	193.6875	14
15	Harvard Medical School	15	1849	126.2667	5

### 3.4 Journals and co-cited journals

A total of 502 academic journals have published papers. **Table 3** presents the top 10 journals, which collectively published 272 papers, representing 19.81% of all publications. “International Journal of Molecular Sciences” leads the list with the highest number of studies published (n=62), closely followed by “Frontiers in Immunology” (n=46), “Frontiers in Pharmacology” (n=47, accounting for 3.59%), and “Frontiers in Endocrinology” (n=22). Notably, “Frontiers in Immunology” stands out with the highest impact factor of 7.3. Among these top 10 journals, seven are positioned in the Q1 quadrant of JCR, while the remaining three are in the Q2 quadrant.

**Table 3:** Top 10 journals related to the Role of NLRP3 infalmmasome in Diabetes.

Rank	Journal	Documents(n)	IF(2022)	JCR(2022)	Country
1	International Iournal Of Molecular Sciences	62	5.6	Q1	USA
2	Frontiers in Immunology	46	7.3	Q1	SWITZERLAND
3	Frontiers in Pharmacology	27	5.6	Q1	SWITZERLAND
4	Frontiers in Endocrinology	22	5.2	Q1	SWITZERLAND
5	International Immunopharmacology	22	5.6	Q2	NETHERLANDS
6	Scientific Reports	21	4.6	Q2	England
7	Antioxidants	19	7	Q1	SWITZERLAND
8	Frontiers in Physiology	19	4	Q2	SWITZERLAND
9	Biomedicine & Pharmacotherapy	17	7.5	Q1	FRANCE
10	Life Sciences	17	6.1	Q1	ENGLAND

Out of 6001 jointly cited journals, 19 have been cited over 1000 times. **Table 4** presents the top 10 most cited journals. With 2845 citations, Diabetes ranks as the most cited journal, closely followed by Nature (2708) and the Journal of Biological Chemistry (2176). Among the top 10 co-cited journals, Nature Medicine leads with the highest impact factor of 82.9, succeeded by Nature (64.8) and Cell (64.5). Notably, 7 out of these 10 commonly cited journals are positioned in the Q1 zone of JCR, while the remaining three are in the JCR Q2 area.

**Table 4:** Top 10 co-cited journals related to the Role of NLRP3 infalmmasome in Diabetes.

Rank	Journal	Ciatation	IF(2022)	JCR(2022)	Country
1	DIABETES	2845	7.7	Q1	USA
2	NATURE	2708	64.8	Q1	ENGLAND
3	JOURNAL OF BIOLOGICAL CHEMISTRY	2176	4.8	Q2	USA
4	PLos One	1906	3.7	Q2	USA
5	PROCEEDINGS OF THE NATIONAL ACADEMY OF SCIENCES OF THE UNITED STATES OF AMERICA	1631	11.1	Q1	USA
6	JOURNAL OF IMMUNOLOGY	1612	4.4	Q2	USA
7	NATURE IMMUNOLOGY	1601	30.5	Q1	USA
8	JOURNAL OF CLINICAL INVESTIGATION	1442	15.9	Q1	USA
9	CELL	1426	64.5	Q1	USA
10	NATURE MEDICINE	1362	82.9	Q1	USA

### 3.5 Authors and co-cited authors

A total of 7859 authors took part in this subject study. **Table 5** highlights the top ten authors and those with the most citations based on the number of articles they’ve published. Notably, Wang Wei lead with 10 articles, followed by Li Xiang with 8, and Wang Yan, Yang Guang, Zhang Li, and Zhou Rongbin, all with 7 articles each. Co-cited authors refer to those frequently cited together in various publications. According to **Table 5**, Zhou Rb, Martinon F, and Schroder K stand out for having the highest number of co-citations.

**Table 5:** Top 10 authors and co-cited authors related to the Role of NLRP3 infalmmasome in Diabetes.

Rank	Authou	Count	Co-cited author	Co-citation
1	wang, wei	10	zhou, rb	346
2	li, xiang	8	martinon, f	294
3	wang, yan	7	schroder, k	284
4	yang, guang	7	vandanmagsar, b	284
5	zhang, li	7	wen, ht	245
6	zhou, rongbin	7	masters, sl	219
7	che, hui	6	hotamisligil, gs	215
8	chen, li	6	lamkanfi, m	210
9	chen, wei	6	donath, my	208
10	chen, yang	6	ridker, pm	208

**Figure 5:**
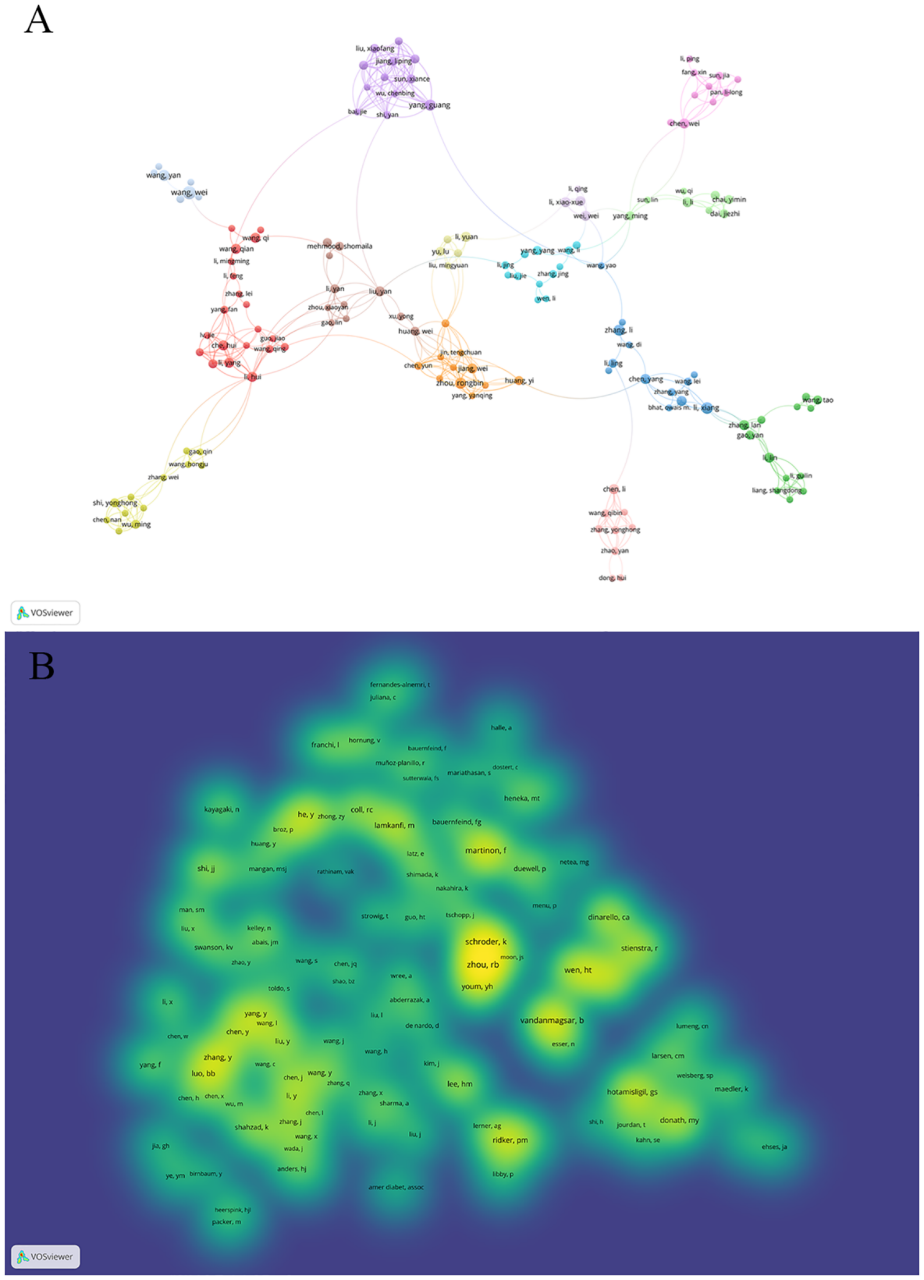
A visualization map of Authors and co-cited authors. **(A)** illustrates the collaboration among the top ten authors. The total linkstrength :926.Items:147.Clusters:14.Links:418. **(B)** demonstrates the aggregation of co-cited authors. The total linkstrength :79271.Items:117.Clusters:3.Links:6434. The color brightness means the frequency of occurrence.

**Figure 5A** illustrates author collaboration. In this visualization, created using vosviewer with a minimum occurrence threshold of 3, we observe 332 authors. The figure features 14 colors, signifying 11 distinct clusters of authors. Notably, authors within the same cluster, such as Zhou Rongbin, Jiang Wei, Chen Yun, Huang Yi, and others, demonstrate active collaboration. Additionally, there are collaborative links between nodes spanning different clusters, involving authors like Wang Wei, Wang Yan, Zhang Peng.

Moving on to **Figure 5B**, it showcases the clustering of co-cited authors. Out of 51072 co-cited authors, 33 have been cited over 100 times. This figure, presented as a density map using vosviewer with a minimum occurrence set to 50, showcases 117 authors. The brightness of each color corresponds to the frequency of citations, with higher frequencies resulting in brighter hues, allowing for clear identification of the most frequently co-cited authors.

### 3.6 Co-citations references and references burst

**Figure 6:**
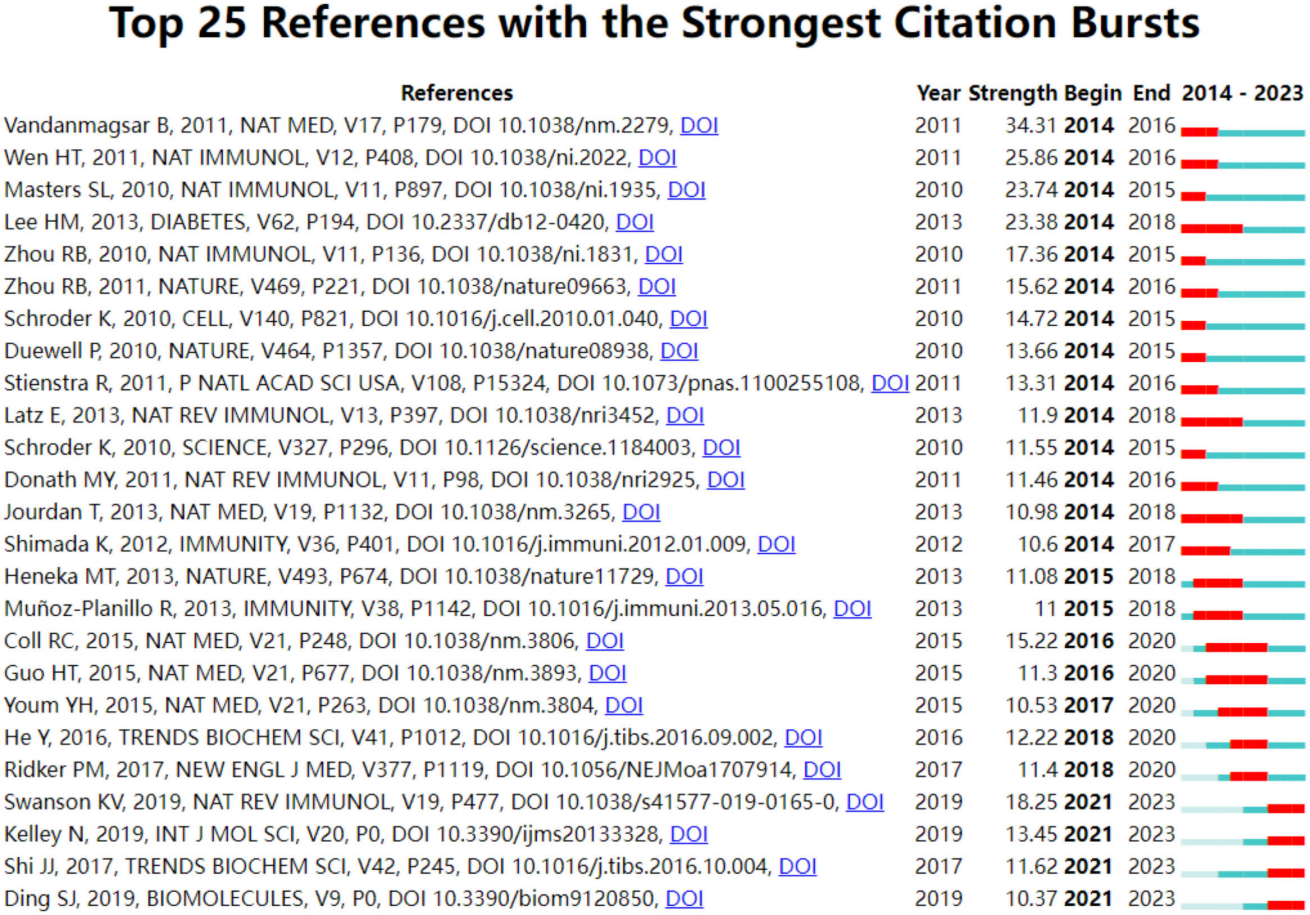
Top 25 references with the strongest citation.

**Table 6** presents the top 10 most commonly cited references. Among these, “NLRP3 inflammasome: molecular activation and regulation to therapeutics” (19) stands out with 100 citations, making it the most frequently cited. This is followed by “NLRP3 inflammasome: an overview of mechanisms of activation and regulation” (20) with 82 citations, and “Upregulated NLRP3 inflammasome activation in patients with type 2 diabetes” (21) with 76 citations. **Figure 6** highlights the top 25 references that have experienced the most significant citation explosions. Notably, the year 2014 (14/25,56%) saw the most citation outbreaks.This is followed by 2021 (4/25,16%). Furthermore, it’s worth mentioning that the citation explosion for 4 references (16%) extended until 2023. Specifically, Bolormaa Vandanmagsar’s article (22) exhibited a strong citation burst between 2014 and 2016, with the highest intensity of 34.31.

**Table 6:** Top 10 co-cited references related to the Role of NLRP3 infalmmasome in Diabetes.

Rank	ID	Title	Co-citation	Centrality
1	Karen V Swanson	The NLRP3 inflammasome: molecular activation and regulation to therapeutics	100	0.02
2	Nathan Kelley	The NLRP3 Inflammasome: An Overview of Mechanisms of Activation and Regulation	82	0.01
3	Hye-Mi Lee	Upregulated NLRP3 Inflammasome Activation in Patients With Type 2 Diabetes	76	0.03
4	Bolormaa Vandanmagsar	The NLRP3 inflammasome instigates obesity-induced inflammation and insulin resistance	74	0
5	Paul M Ridker	Antiinflammatory Therapy with Canakinumab for Atherosclerotic Disease	64	0.06
6	Rebecca C Coll	A small-molecule inhibitor of the NLRP3 inflammasome for the treatment of inflammatory diseases	63	0.12
7	Matthew S J Mangan	Targeting the NLRP3 inflammasome in inflammatory diseases	57	0.05
8	Haitao Wen	Fatty acid–induced NLRP3-ASC inflammasome activation interferes with insulin signaling	56	0.04
9	Yuan He	Mechanism and Regulation of NLRP3 Inflammasome Activation	52	0.02
10	Yang Yang	Recent advances in the mechanisms of NLRP3 inflammasome activation and its inhibitors	47	0.01

### 3.7 Keywords

**Figure 7:**
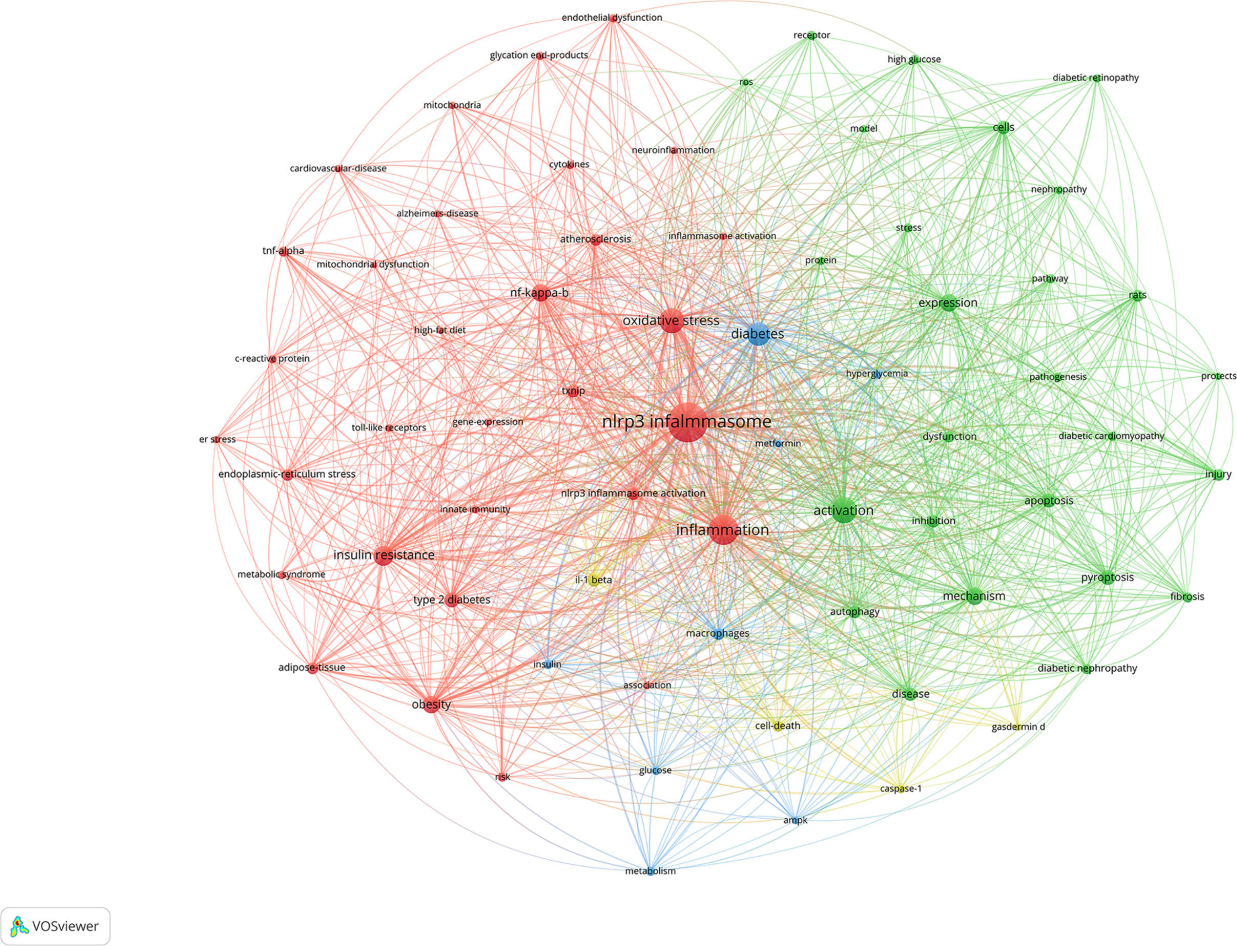
A visualization map of keywords.The total linkstrength :16211.Items:69.Clusters:4.Links:1923.

**Figure 7** illustrates the clustering diagram derived from the keyword co-occurrence analysis. Prior to conducting the analysis, we meticulously organized the keywords, making the following replacements: “nlrp3”, “nalp3 inflammasome” replace by “NLRP3 infalmmasome”; “inflammasome”, “inflammasomes” replace by “inflammation”; “type-2-diabetes-mellitus”, “type-2 diabetes-mellitus”, “type 2 diabetes mellitus” replace by “type 2 diabetes”; “insulin-resistance” replace by “insulin resistance”; “diabetes mellitus” “diabetes-mellitus”, “mellitus” replace by “diabetes”; “mechanisms” replace by “mechanism”; “mice” replace by “rat”. We set a minimum occurrence threshold of 30, through which we filtered a total of 69 keywords. Subsequently, we computed the co-occurrence intensity among these keywords. Based on their frequency, the top 10 keywords are: “ NLRP3 infalmmasome”, “inflammation”, “activation”, “oxidative stress”, “diabetes”, “insulin resistance”, “nf-kappa-b”, “expression”, “mechanism”, and ” obesity”.

**Table 7** presents the top 10 keywords ranked by frequency, among which five are grouped within the red cluster. These keywords are classified into four distinct categories: first, the association between inflammasome and inflammatory diseases related to DM; second, the exploration of the pathogenesis of DM-related diseases at the cellular level; third, the characteristics of DM and its associated suppression methods; and fourth, pyroptosis and other morphological changes in cells.

**Table 7:** Co-occurrence analysis of keywords and top 10 keywords in the 4 clusters.

Cluster	Color	Keywords(occurences)
1	red	NLRP3 infalmmasome(879) Inflammation(514) oxidative stress(357) insulin resistance(231) nf-kappa-b(182) obesity(172) type 2diabetes(134) txnip(93) nlrp3 inflammasome activation(88) adipose-tissue(80) atherosclerosis(76) endoplasmic-reticulum stress(76) tnf-alpha(61) risk(48) metabolic syndrome(44) c-reactive protein(40) endothelial dysfunction(40) cytokines(39) glycation end-products(39) alzheimers-disease(37) toll-like receptors(37) cardiovascular-disease(35) innate immunity(34) association(33) inflammasomeactivation(33) mitochondria(33) er stress(32) gene-expression(31) high-fat diet(31) neuroinflammation(31) mitochondrial dysfunction(30)
2	green	Activation(383) expression(177) mechanism(172) pyroptosis(131) apoptosis(111) cells(105) disease(99) inhibition(91) autophagy(86) rats(82) injury(75) dysfunction(65) diabeticnephropathy(63) fibrosis(61) diabeticcardiomyopathy(52) highglucose(51) Pathogenesis(49) pathway(49) receptor(48) stress(46) protein(45) diabeticretinopathy(42) nephropathy(42) ros(36) model(33) protects(31)
3	blue	Diabetes(305) macrophages(78) insulin(49) metabolism(49) hyperglycemia(44) glucose(39) metformin(32) ampk(31)
4	yellow	il-1 beta(86) cell-death(70) caspase-1(42) gasdermin d(33)

**Figure 8:**
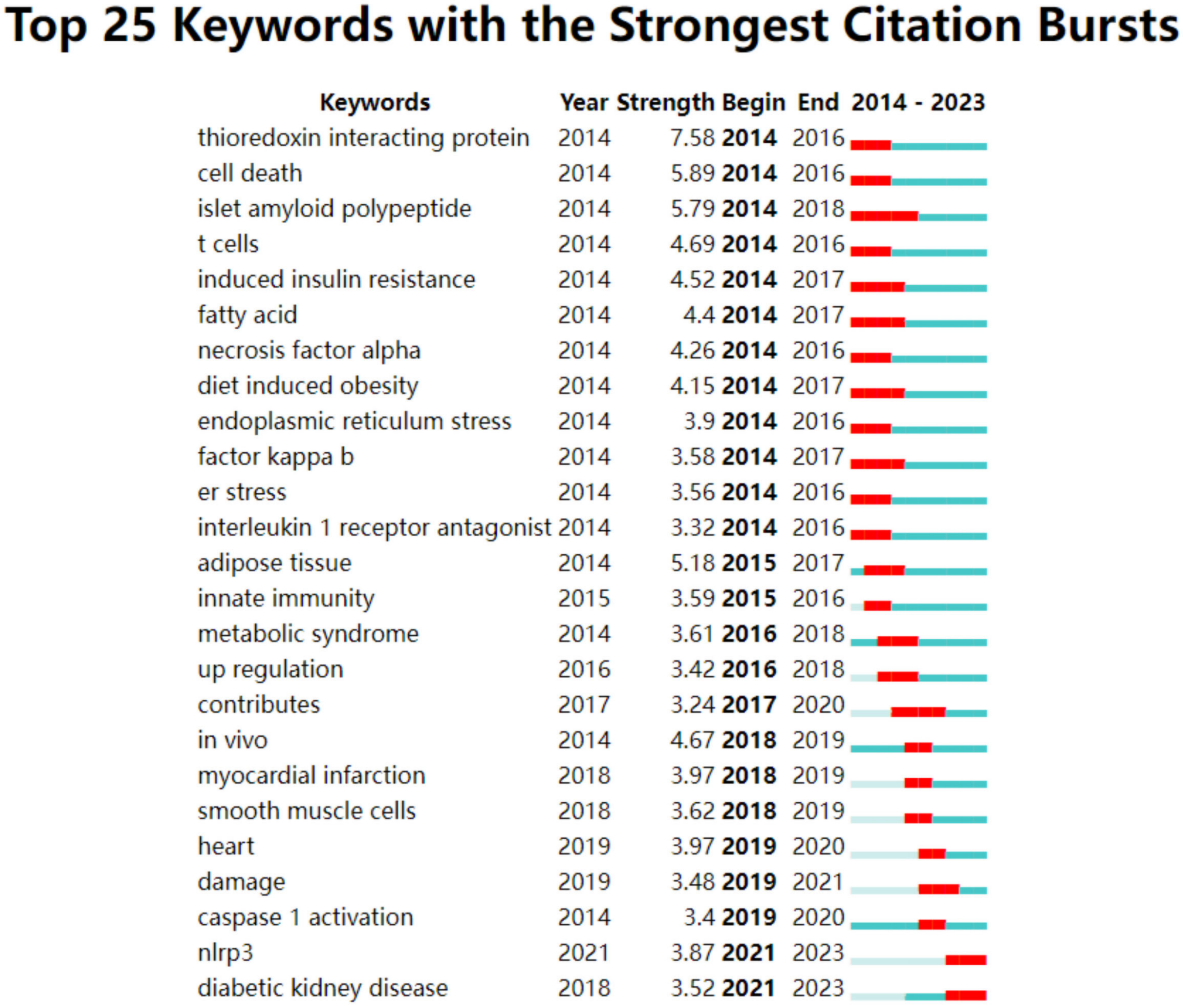
Top 25 keywords with the strongest citation.

**Figure 8** illustrates the 25 keywords that exhibited the most significant outbreaks. The year that witnessed the highest frequency of keyword outbreaks was 2014 (12/25,48%), followed by 2018(3/25,12%) and 2019(3/25,12%). It’s notable that” NLRP3 “and “diabetic kidney disease” have persisted in breaking out up until 2023.

## 4 Discussion

### 4.1 General information discussion

In this study, vosviewer, CiteSpace, and Scimago Graphica were utilized to perform scientometrics analysis. We collected scientific literature exploring the role of NLRP3 infalmmasome in DM, spanning from 2014 to 2023. By 2023, a total of 1373 documents were identified. The research yielded substantial results, culminating in a peak of published papers in 2022. The volume of papers published during each period offers insight into the evolving research trends in this field. Prior to 2019, articles on this topic were scarce and demonstrated sluggish growth. Notably, Karen v Swanson’s (19) 2019 article emerged as the most cited, delving into the molecular, cell biology, and biochemical foundations of NLRP3 infalmmasome activation and regulation. That same year, Nathan Kelley (20) provided a comprehensive overview of NLRP3 infalmmasome’s activation mechanism, encompassing various signal transduction events, post-translational modifications, and the modulation of its interacting partners. Since then, the rapid escalation in published papers underscores the soaring research interest in this domain. The sustained increase in publications through 2023 highlights the significant research value and promising development potential of this topic.

The number of publications and citation frequency stand as significant metrics for assessing the scientific research prowess of a country, region, or institution. This particular research field has seen contributions from 78 countries, with China leading in the total number of publications and citations. Germany boasts the highest average citation count, while the United States demonstrates the most robust connectivity with other nations. From a temporal perspective, the United States, Germany, and other developed countries have made notable contributions to this field. However, over time, several developing countries have emerged in this field, offering a source of potential inspiration for researchers in those regions. Notably, the top five most productive institutions hail from China. When compared to enterprises, research institutes, and social research organizations, university research teams exhibit clear advantages in this domain. The top five universities are all prestigious institutions directly affiliated with the Ministry of Education of the China. Nonetheless, there is room for improvement in inter-university collaboration. It is recommended that universities enhance cooperation, facilitating the exchange of research teams and fostering cross-regional and inter-institutional interactions.

Journal analysis reveals that the international journal of Molecular Sciences has published the most research on this topic. Meanwhile, Frontiers holds the highest proportion among the top 10 journals (4/10, 40%), highlighting their notable contributions in disseminating research on this subject. Upon examining the top 10 most-cited journals, it is evident that 90% originate from the United States, reflecting the significant influence the country holds in this research field. Notably, high-impact journals such as Nature and Cell are included in this list, thus providing valuable guidance for future related studies.

Based on the author co-occurrence chart (refer to **Figure 5A**) derived from author data, it is evident that among the top 10 authors with the highest number of published articles, all originate from Chinese institutions. Notably, Wang Wei and Wang Yan exhibited extensive collaboration, as did Li Xiang, Zhang Li, and Chen Yang, while the remaining high-output authors operated independently. This pattern mirrors the institutional analysis structure. We recommend enhancing collaboration and exchange among authors to broaden the scope of research. Simultaneously, we advise authors or institutions engaged in related studies to foster stronger ties and collaborative efforts.

Among the top 10 most cited authors, several distinct clustering patterns emerge. Zhou Rb, Martinon F, Schroder K, and Lamkanfi M form one cluster, while Vandanmagsar B, Wen HT, Masters SL, Hotamisligil GS, Donath MY, and Ridker PM constitute another. The clustering amongst these cited authors is pronounced. Of particular note is Professor Zhou Rb from the School of Basic Medicine, Department of Life Sciences and Medicine at the University of Science and Technology of China, who features prominently in both publishing and citation metrics. Professor Zhou pioneered the discovery of the critical role played by the danger signal sensing receptor NLRP3 infalmmasome in sterile inflammation and disease progression, specifically in complex chronic conditions like T2DM. His work also uncovered the primary mechanism behind NLRP3 infalmmasome activation (23, 24). In 2015, his team further elucidated the endogenous mechanism of inflammasome regulation in cells, introducing the concept that the neurotransmitter dopamine suppresses inflammasome activation via the dopamine D1 receptor. This groundbreaking research offers a potential therapeutic target for NLRP3-driven diseases and has been cited 432 times (25).

### 4.2 Knowledge base analysis

The following presents an analysis of the top 10 most cited literatures:

In 2019, Karen V Swanson published an article in Nat Rev Immunol entitled “The NLRP3 inflammasome: molecular activation and regulation to therapeutics” (19). This article stands as the most frequently cited literature on this research topic. It delves into the activation of NLRP3 infalmmasome and explores its targeted regulation from molecular, cellular biological, and biochemical viewpoints. The piece offers fresh perspectives and insights for the treatment of NLRP3 infalmmasome. However, this literature does not explain in detail the activation process of NLRP3 inflammasome in different disease contexts. In addition, an in-depth exploration of the differences in efficacy in different disease states may be lacking for the use of NLRP3 inflammasome in therapy.

Nathan Kelley’s “The NLRP3 Inflammasome: An Overview of Mechanisms of Activation and Regulation” (20), published in Int J Mol Sci in 2019, stands as the second most frequently cited article on this research topic. In this piece, he elucidated how abnormal activation of NLRP3 infalmmasome is linked to a wide range of inflammatory diseases, including DM. Furthermore, he delved into different methods of stimulating inflammasome activation and examined the assembly process of the inflammasome post-activation, thereby paving a new path for inhibiting the inflammasome and advancing the treatment of inflammatory diseases like DM. The shortcoming is the incomplete description of the mechanisms of NLRP3 inflammasome activation and regulation.

The third article, published by Hye-Mi Lee in 2013, is titled “Upregulated NLRP3 Inflammasome Activation in Patients with Type 2 Diabetes” (21). This article explores the clinical correlation between the activation of NLRP3 infalmmasome and the pathogenesis of T2DM, offering a fresh approach for the treatment of various metabolic and inflammatory ailments, including diabetes mellitus. The literature has not fully explored the causal relationship between NLRP3 inflammasome activation and T2DM, and how this activation affects diabetes progression and response to therapy.

The article titled “The NLRP3 Inflammasome Instigates Obesity-Induced Inflammation and Insulin Resistance” (22), was authored by Bolormaa Vandanmagsarin and published in NAT MED in 2011. It secured the fourth spot on the list of CO-cited literatures and topped the list in terms of the highest emergence intensity. Through clinical and animal experiments, this study established that in obese T2DM patients, the decrease in NLRP3 infalmmasome adipose tissue expression correlates with reduced inflammation and enhanced insulin sensitivity. This study offers compelling evidence for the involvement of NLRP3 infalmmasome in T2DM, serving as a valuable reference for further exploration by researchers. But there is no detailed discussion of the specific molecular pathways of how obesity triggers inflammation and insulin resistance through the NLRP3 inflammasome, and how these pathways affect the treatment of obesity and related metabolic diseases.

The fifth article, authored by Paul M. Ridker and titled “Antiinflammatory Therapy with Canakinumab for Atherosclerotic Disease” (26), was published in the New England Journal of Medicine in 2017. Based on clinical trials, the article suggests that inhibiting the function of NLRP3 infalmmasome could potentially reduce cardiovascular risk. This literature focuses on the anti-inflammatory effects of Canakinumab, but does not fully explore its direct effect on the NLRP3 inflammasome and how this effect affects the progression of atherosclerosis.

Rebecca C. coll published an article in Nature Medicine in 2015 (27), discussing a small molecule inhibitor of the NLRP3 inflammasome for the treatment of inflammatory diseases. In this article, the author also put forth the idea that NLRP3 infalmmasome plays a part in the inflammatory process, which abnormally triggers the pathogenic factors of inflammatory diseases, including T2DM. Simultaneously, the study confirmed that the inhibitor of NLRP3 infalmmasome holds potential as a treatment for related autoinflammatory and autoimmune diseases, thus paving a new path for further exploring the significance of NLRP3 infalmmasome in human health and disease. The literature may not provide sufficient data to demonstrate the specificity and potency of small molecule inhibitors, as well as their efficacy and safety in different inflammatory diseases.

Matthew J. Mangan published “Targeting the NLRP3 Inflammasome in Inflammatory Diseases” (28) in Nat Rev Drug Discov in 2018. This paper provides a comprehensive summary of the structure and activation mechanism of NLRP3 infalmmasome, and delves deeper into the discovery and development of new therapies that target this specific entity. However, there is no detailed discussion of the differences in the role of the NLRP3 inflammasome in different inflammatory diseases, as well as the therapeutic efficacy and potential side effects of targeting the NLRP3 inflammasome.

In 2011, Haitao Wen published an article titled “Fatty Acid-Induced NLRP3-ASC Inflammasome Activation Interactions with Insulin Signaling” (29) in NAT Immunol. The article presented a novel hypothesis that saturated fatty acid palmitate, unlike unsaturated oleate, has the potential to trigger the activation of the NLRP3-ASC inflammasome. This activation subsequently results in the production of Caspase-1, IL-1β, and IL-18, which consequently diminishes glucose tolerance and insulin sensitivity. This research offers a fresh insight into the connection between inflammation, diet, and end-stage DM. The literature has not fully explored the specific mechanisms of how fatty acids affect insulin signalling through the NLRP3 inflammasome, and how this effect may be linked to the development of metabolic disease.

The article ranked No. 9 is “Mechanism and Regulation of NLRP3 Inflammasome Activation” (30), authored by Yuan He and published in Trends Biochem Sci in 2016. This study provides a comprehensive overview of the current understanding of the activation mechanism and regulation of NLRP3 infalmmasome, alongside recent advancements in non-canonical and alternative inflammasome pathways. This literature does not cover recent research advances in the mechanisms of NLRP3 inflammasome activation and regulation, especially the changes in mechanisms in different disease contexts.

The 10th most cited document, published by Yang Yang in Cell Death DIS in 2019, highlights recent advancements in the mechanisms of NLRP3 inflammasome activation and its inhibitors (31). This document predominantly summarizes the current understanding of the regulation mechanism behind NLRP3 infalmmasome activation, alongside inhibitors that specifically and directly target NLRP3 infalmmasome. As a review article, there may be a lack of coverage of the latest research findings, resulting in an incomplete or updated description of the latest advances in NLRP3 inflammasome activation mechanisms and inhibitors.

Generally, the top 10 co-cited literatures delved into NLRP3 infalmmasome and DM, with a specific focus on T2DM, through reviews, clinical trials, and animal experiments. This comprehensive approach established a theoretical foundation for conducting relevant research on multiple levels and from various perspectives. Although there are some limitations, it suggests directions for future research, including an in-depth exploration of the specific mechanism of action of the NLRP3 inflammasome in different diseases and the development of more potent inhibitors of the NLRP3 inflammasome.

### 4.3 Hot topics and introductory discussion

The abrupt emergence of CO-cited literature serves as an indicator of prevalent research trends within a given timeframe. Prior to the outbreak, among the 25 referenced studies, 11 delved into the activation of NLRP3 infalmmasome, exploring its signaling pathways, assembly, regulation, and other related aspects (19, 20, 26, 30, 32–38). Meanwhile, 10 papers examined the significance of NLRP3 infalmmasome in the progression of T2DM, covering perspectives such as inflammation, insulin sensitivity, and macrophage involvement (21, 22, 27, 29, 39–44). One study specifically addressed the role of NLRP3 infalmmasome in obesity and insulin resistance (45), while two others investigated its function in activating il-β related to Alzheimer’s disease and atherosclerosis (46, 47). Currently, four articles are at the forefront, highlighting present and future research trajectories, primarily centered around the activation mechanism of the inflammasome, its connection to pyroptosis (48), and its regulatory role in DM. Evidently, the activation, regulation, and impact of the inflammasome in DM have emerged as a significant focus of numerous scholars over the past decade.

Keywords stand as the focal point of any article. The concurrent emergence and surge of specific keywords can reveal trends in research hotspots and serve as an introduction to the topic. Within the keyword co-occurrence cluster, a primary category emerges: the association between inflammasome, DM, and other inflammatory diseases. Consuming a high-fat diet elevates the levels of free fatty acids and endotoxins, such as lipopolysaccharide, in the bloodstream. This, in turn, activates PAMPs or dampens the Toll-like receptor signaling pathway, facilitating downstream nf-κB transcription and boosting the expression of NLRP3 infalmmasome protein. Chronic endoplasmic reticulum stress and the activation of the unfolded protein response, triggered by either endogenous or exogenous injuries, have the potential to disrupt calcium and redox homeostasis. This disruption can lead to oxidative stress due to protein overload, ultimately causing mitochondrial dysfunction. As calcium is released from the endoplasmic reticulum, it stimulates the production of mitochondrial reactive oxygen species. The toxic accumulation of reactive oxygen species within the endoplasmic reticulum and mitochondria disrupts fundamental organelle functions, resulting in the activation of NLRP3 infalmmasome. Furthermore, advanced glycation end products emerge as critical factors in inflammation, given their interconnectedness with immunity, aging, mitochondrial dysfunction, and inflammasome activation. Notably, inflammatory expression is intimately linked to insulin resistance, obesity, T2DM, atherosclerosis, and Alzheimer’s disease.

The second objective is to investigate the pathogenesis of diseases related to DM at the cellular level. NLRP3 infalmmasome holds a strong correlation with pyroptosis. According to Quanwei Li (49) and colleagues, endoplasmic reticulum stress facilitates pyroptosis via the nf-κb/nlrp3 pathway, subsequently altering the pathological progression of diabetic nephropathy. Pan Liu (50) and co-authors provided a comprehensive overview of the molecular mechanisms, associated pathways, and targeted therapies involved in pyroptosis. They posited that pyroptosis in diabetic cardiomyopathy is mediated by the nf-κB signaling pathway, the mtROS-mediated signaling pathway, and several other signaling pathways. Yimeng Sun (51) and associates established that the formation of inflammasomes in innate immune cells and the subsequent induction of pyroptosis can trigger inflammation in various retinal diseases. The monitoring of pyroptosis shows promise for clinical application in the early diagnosis, management, and prognosis prediction of retinal diseases.

By examining the third category, we gain an understanding that DM frequently presents with hyperglycemia, insulin abnormalities, metabolic irregularities, and various other symptoms. These can be modulated by AMPK and other associated signaling pathways. The fourth category predominantly highlights key proteins and expressions that are commonly observed during pyroptosis.

This study identified 25 emergent words that surfaced between 2014 and 2023, reflecting the latest trends and abrupt shifts in current research hotspots. Referring to **Figure 8**, the past five years have seen a focus on heart, damage, caspase 1 activation, NLRP3, and diabetic kidney disease. Researchers have not just probed into the inflammasome but also conducted extensive studies on the related ailments stemming from DM. Furthermore, pyroptosis, diabetic cardiomyopathy, and diabetic nephropathy have garnered significant attention since 2019, thereby guiding relevant researchers in their explorations.

## 5 Limitations

This study primarily focused on the most commonly used WoS database, leading to a possible omission of relevant articles from other databases. Furthermore, our scope was limited to English-language articles, excluding high-quality non-English studies. Additionally, we only included articles and reviews, disregarding political and social publications such as editorials and books. While our search strategy was extensive, there is a possibility that other pertinent keywords may have been overlooked, potentially impacting our findings. Nonetheless, considering the vast amount of data generated from the numerous publications included in this study, any potential bias is expected to be minimal.

## 6 Conclusion

In this study, we conducted a scientometrics evaluation of publications exploring the role of NLRP3 infalmmasome in DM, focusing on the period from 2014 to 2023. Our analysis reveals a rapid surge in document production related to this topic since 2019. Among the nations contributing to this body of literature, China stands out as the most prolific publisher. Notably, China and the United States emerge as the two countries with the strongest collaborative efforts. Harbin Medical University, Nanjing Medical University, and Central South University lead in terms of publication volume. “International Journal of Molecular Sciences” boasts the highest number of relevant research papers on the subject. Regarding authorship, “NLRP3 inflammasome: molecular activation and regulation to therapeutics” stands out as the most frequently cited work with 100 citations. Furthermore, keyword analysis highlights the need for further exploration of NLRP3 infalmmasome’s role in DM, particularly in T2DM and diabetes-related diseases. Given the significant burden of DM in today’s world, there is a growing interest in improving DM outcomes through targeted regulation of NLRP3 infalmmasome, and this study aims to facilitate further advancements in this critical field.

## References

[1] Home, Resources, diabetes L with, Acknowledgement, FAQs, Contact, Policy P. IDF Diabetes Atlas. Available online at: https://diabetesatlas.org/atlas/fourth-edition/ (Accessed November 13, 2024).

[2] Home, Resources, diabetes L with, Acknowledgement, FAQs, Contact, Policy P. IDF Diabetes Atlas. Available online at: https://diabetesatlas.org/atlas/fifth-edition/ (Accessed November 13, 2024).

[3] Home, Resources, diabetes L with, Acknowledgement, FAQs, Contact, Policy P. IDF Diabetes Atlas. Available online at: https://diabetesatlas.org/atlas/sixth-edition/ (Accessed November 13, 2024).

[4] Home, Resources, diabetes L with, Acknowledgement, FAQs, Contact, Policy P. IDF Diabetes Atlas. Available online at: https://diabetesatlas.org/atlas/seventh-edition/ (Accessed November 13, 2024).

[5] Home, Resources, diabetes L with, Acknowledgement, FAQs, Contact, Policy P. IDF Diabetes Atlas. Available online at: https://diabetesatlas.org/atlas/eighth-edition/ (Accessed November 13, 2024).

[6] Saeedi P Petersohn I Salpea P Malanda B Karuranga S Unwin N. Global and regional diabetes prevalence estimates for 2019 and projections for 2030 and 2045: Results from the International Diabetes Federation Diabetes Atlas, 9th edition. Diabetes Res Clin Pract. (2019) 157:107843. doi: 10.1016/j.diabres.2019.107843 31518657

[7] Home, Resources, diabetes L with, Acknowledgement, FAQs, Contact, Policy P. IDF Diabetes Atlas 2021. Available online at: https://diabetesatlas.org/atlas/tenth-edition/ (Accessed November 14, 2024). IDF Diabetes Atlas.

[8] Yu M Ning FTE Liu C Liu Y-C. Interconnections between diabetic corneal neuropathy and diabetic retinopathy: diagnostic and therapeutic implications. Neural Regener Res. (2025) 20:2169–80. doi: 10.4103/NRR.NRR-D-24-00509 PMC11759029 39359077

[9] Dixit A Apte MM Tupe RS. Syzygium cumini (L.) skeels mitigate diabetic nephropathy by regulating Nrf2 pathway and mitocyhondrial dysfunction: *In vitro* and *in vivo* studies. J Ethnopharmacol. (2025) 336:118684. doi: 10.1016/j.jep.2024.118684 39127117

[10] Shan S Luo Z Yao L Zhou J Wu J Jiang D. Cross-country inequalities in disease burden and care quality of chronic kidney disease due to type 2 diabetes mellitus, 1990-2021: Findings from the global burden of disease study 2021. Diabetes Obes Metab. (2024) 26:5950–9. doi: 10.1111/dom.15969 39344843

[11] Rohm TV Meier DT Olefsky JM Donath MY. Inflammation in obesity, diabetes, and related disorders. Immunity. (2022) 55:31–55. doi: 10.1016/j.immuni.2021.12.013 35021057 PMC8773457

[12] Romero A Dongil P Valencia I Vallejo S Hipólito-Luengo ÁS Díaz-Araya G. Pharmacological blockade of NLRP3 inflammasome/IL-1β-positive loop mitigates endothelial cell senescence and dysfunction. Aging Dis. (2022) 13:284–97. doi: 10.14336/AD.2021.0617 PMC8782550 35111374

[13] Ren W Sun Y Zhao L Shi X. NLRP3 inflammasome and its role in autoimmune diseases: A promising therapeutic target. BioMed Pharmacother Biomed Pharmacother. (2024) 175:116679. doi: 10.1016/j.biopha.2024.116679 38701567

[14] Satheesan A Kumar J Leela KV Murugesan R Chaithanya V Angelin M. Review on the role of nucleotide-binding oligomerization domain-like receptor protein 3 (NLRP3) inflammasome pathway in diabetes: mechanistic insights and therapeutic implications. Inflammopharmacology. (2024) 32:2753–79. doi: 10.1007/s10787-024-01556-2 39160391

[15] Ye T Tao W-Y Chen X-Y Jiang C Di B Xu L-L. Mechanisms of NLRP3 inflammasome activation and the development of peptide inhibitors. Cytokine Growth Factor Rev. (2023) 74:1–13. doi: 10.1016/j.cytogfr.2023.09.007 37821254

[16] van Eck NJ Waltman L. Software survey: VOSviewer, a computer program for bibliometric mapping. Scientometrics. (2010) 84:523–38. doi: 10.1007/s11192-009-0146-3 PMC2883932 20585380

[17] Chen C Song M. Visualizing a field of research: A methodology of systematic scientometric reviews. PloS One. (2019) 14:e0223994. doi: 10.1371/journal.pone.0223994 31671124 PMC6822756

[18] Chen C. A glimpse of the first eight months of the COVID-19 literature on microsoft academic graph: themes, citation contexts, and uncertainties. Front Res Metr Anal. (2020) 5:607286. doi: 10.3389/frma.2020.607286 33870064 PMC8025977

[19] Swanson KV Deng M Ting JP-Y. The NLRP3 inflammasome: molecular activation and regulation to therapeutics. Nat Rev Immunol. (2019) 19:477–89. doi: 10.1038/s41577-019-0165-0 PMC7807242 31036962

[20] Kelley N Jeltema D Duan Y He Y. The NLRP3 inflammasome: an overview of mechanisms of activation and regulation. Int J Mol Sci. (2019) 20:3328. doi: 10.3390/ijms20133328 31284572 PMC6651423

[21] Lee H-M Kim J-J Kim HJ Shong M Ku BJ Jo E-K. Upregulated NLRP3 inflammasome activation in patients with type 2 diabetes. Diabetes. (2013) 62:194–204. doi: 10.2337/db12-0420 23086037 PMC3526026

[22] Vandanmagsar B Youm Y-H Ravussin A Galgani JE Stadler K Mynatt RL. The NALP3/NLRP3 inflammasome instigates obesity-induced autoinflammation and insulin resistance. Nat Med. (2011) 17:179–88. doi: 10.1038/nm.2279 PMC3076025 21217695

[23] Huang Y Xu W Zhou R. NLRP3 inflammasome activation and cell death. Cell Mol Immunol. (2021) 18:21149–27. doi: 10.1038/s41423-021-00740-6 PMC8429580 34321623

[24] Wang X Jiang W Yan Y Gong T Han J Tian Z. RNA viruses promote activation of the NLRP3 inflammasome through a RIP1-RIP3-DRP1 signaling pathway. Nat Immunol. (2014) 15:1126–33. doi: 10.1038/ni.3015 25326752

[25] Yan Y Jiang W Liu L Wang X Ding C Tian Z. Dopamine controls systemic inflammation through inhibition of NLRP3 inflammasome. Cell. (2015) 160:62–73. doi: 10.1016/j.cell.2014.11.047 25594175

[26] Ridker PM Everett BM Thuren T MacFadyen JG Chang WH Ballantyne C. Antiinflammatory therapy with canakinumab for atherosclerotic disease. N Engl J Med. (2017) 377:1119–31. doi: 10.1056/NEJMoa1707914 28845751

[27] Coll RC Robertson AAB Chae JJ Higgins SC Muñoz-Planillo R Inserra MC. A small-molecule inhibitor of the NLRP3 inflammasome for the treatment of inflammatory diseases. Nat Med. (2015) 21:248–55. doi: 10.1038/nm.3806 PMC4392179 25686105

[28] Mangan MSJ Olhava EJ Roush WR Seidel HM Glick GD Latz E. Targeting the NLRP3 inflammasome in inflammatory diseases. Nat Rev Drug Discovery. (2018) 17:588–606. doi: 10.1038/nrd.2018.97 30026524

[29] Wen H Gris D Lei Y Jha S Zhang L Huang MT-H. Fatty acid-induced NLRP3-ASC inflammasome activation interferes with insulin signaling. Nat Immunol. (2011) 12:408–15. doi: 10.1038/ni.2022 PMC4090391 21478880

[30] He Y Hara H Núñez G. Mechanism and regulation of NLRP3 inflammasome activation. Trends Biochem Sci. (2016) 41:1012–21. doi: 10.1016/j.tibs.2016.09.002 PMC5123939 27669650

[31] Yang Y Wang H Kouadir M Song H Shi F. Recent advances in the mechanisms of NLRP3 inflammasome activation and its inhibitors. Cell Death Dis. (2019) 10:128. doi: 10.1038/s41419-019-1413-8 30755589 PMC6372664

[32] Zhou R Yazdi AS Menu P Tschopp J. A role for mitochondria in NLRP3 inflammasome activation. Nature. (2011) 469:221–5. doi: 10.1038/nature09663 21124315

[33] Latz E Xiao TS Stutz A. Activation and regulation of the inflammasomes. Nat Rev Immunol. (2013) 13:397–411. doi: 10.1038/nri3452 23702978 PMC3807999

[34] Muñoz-Planillo R Kuffa P Martínez-Colón G Smith BL Rajendiran TM Núñez G. K^+^ efflux is the common trigger of NLRP3 inflammasome activation by bacterial toxins and particulate matter. Immunity. (2013) 38:1142–53. doi: 10.1016/j.immuni.2013.05.016 PMC3730833 23809161

[35] Shimada K Crother TR Karlin J Dagvadorj J Chiba N Chen S. Oxidized mitochondrial DNA activates the NLRP3 inflammasome during apoptosis. Immunity. (2012) 36:401–14. doi: 10.1016/j.immuni.2012.01.009 PMC3312986 22342844

[36] Schroder K Tschopp J. The inflammasomes. Cell. (2010) 140:821–32. doi: 10.1016/j.cell.2010.01.040 20303873

[37] Youm Y-H Nguyen KY Grant RW Goldberg EL Bodogai M Kim D. The ketone metabolite β-hydroxybutyrate blocks NLRP3 inflammasome-mediated inflammatory disease. Nat Med. (2015) 21:263–9. doi: 10.1038/nm.3804 PMC4352123 25686106

[38] Zhou R Tardivel A Thorens B Choi I Tschopp J. Thioredoxin-interacting protein links oxidative stress to inflammasome activation. Nat Immunol. (2010) 11:136–40. doi: 10.1038/ni.1831 20023662

[39] Masters SL Dunne A Subramanian SL Hull RL Tannahill GM Sharp FA. Activation of the Nlrp3 inflammasome by islet amyloid polypeptide provides a mechanism for enhanced IL-1β in type 2 diabetes. Nat Immunol. (2010) 11:897–904. doi: 10.1038/ni.1935 20835230 PMC3103663

[40] Jourdan T Godlewski G Cinar R Bertola A Szanda G Liu J. Activation of the Nlrp3 inflammasome in infiltrating macrophages by endocannabinoids mediates beta cell loss in type 2 diabetes. Nat Med. (2013) 19:1132–40. doi: 10.1038/nm.3265 PMC4050982 23955712

[41] Guo H Callaway JB Ting JP-Y. Inflammasomes: mechanism of action, role in disease, and therapeutics. Nat Med. (2015) 21:677–87. doi: 10.1038/nm.3893 PMC4519035 26121197

[42] Ding S Xu S Ma Y Liu G Jang H Fang J. Modulatory mechanisms of the NLRP3 inflammasomes in diabetes. Biomolecules. (2019) 9:850. doi: 10.3390/biom9120850 31835423 PMC6995523

[43] Schroder K Zhou R Tschopp J. The NLRP3 inflammasome: a sensor for metabolic danger? Science. (2010) 327:296–300. doi: 10.1126/science.1184003 20075245

[44] Donath MY Shoelson SE. Type 2 diabetes as an inflammatory disease. Nat Rev Immunol. (2011) 11:98–107. doi: 10.1038/nri2925 21233852

[45] Stienstra R van Diepen JA Tack CJ Zaki MH van de Veerdonk FL Perera D. Inflammasome is a central player in the induction of obesity and insulin resistance. Proc Natl Acad Sci U.S.A. (2011) 108:15324–9. doi: 10.1073/pnas.1100255108 PMC3174591 21876127

[46] Duewell P Kono H Rayner KJ Sirois CM Vladimer G Bauernfeind FG. NLRP3 inflammasomes are required for atherogenesis and activated by cholesterol crystals. Nature. (2010) 464:1357–61. doi: 10.1038/nature08938 PMC2946640 20428172

[47] Heneka MT Kummer MP Stutz A Delekate A Schwartz S Vieira-Saecker A. NLRP3 is activated in Alzheimer’s disease and contributes to pathology in APP/PS1 mice. Nature. (2013) 493:674–8. doi: 10.1038/nature11729 PMC3812809 23254930

[48] Shi J Gao W Shao F. Pyroptosis: gasdermin-mediated programmed necrotic cell death. Trends Biochem Sci. (2017) 42:245–54. doi: 10.1016/j.tibs.2016.10.004 27932073

[49] Li Q Zhang K Hou L Liao J Zhang H Han Q. Endoplasmic reticulum stress contributes to pyroptosis through NF-κB/NLRP3 pathway in diabetic nephropathy. Life Sci. (2023) 322:121656. doi: 10.1016/j.lfs.2023.121656 37011874

[50] Liu P Zhang Z Chen H Chen Q. Pyroptosis: Mechanisms and links with diabetic cardiomyopathy. Ageing Res Rev. (2024) 94:102182. doi: 10.1016/j.arr.2023.102182 38182080

[51] Sun Y Li F Liu Y Qiao D Yao X Liu G-S. Targeting inflammasomes and pyroptosis in retinal diseases-molecular mechanisms and future perspectives. Prog Retin Eye Res. (2024) 101:101263. doi: 10.1016/j.preteyeres.2024.101263 38657834

